# Development of seam tracking device in asynchronous tandem welding with arc sensing

**DOI:** 10.1038/s41598-022-23299-2

**Published:** 2022-11-03

**Authors:** Bo Wook Seo, Dong-Yoon Kim, Cheolhee Kim, Seok Kim, Young Tae Cho

**Affiliations:** 1 Department of Smart Manufacturing Engineering, Changwon National University, Changwon, 51140 Republic of Korea; 2 Department of Mechanical Engineering, Changwon National University, Changwon, 51140 Republic of Korea; 3 Advanced Joining and Additive Manufacturing R&D Department, Korea Institute of Industrial Technology, 156 Gaetbeol-Ro (Songdo-Dong), Yeonsu-Gu, Incheon, 21999 Republic of Korea

**Keywords:** Engineering, Mechanical engineering

## Abstract

Tandem welding is extensively used for welding large structures, such as ships and plants, for increased welding speed and volume. Seam tracking is essential because of a large amount of thermal deformation. However, in tandem welding, arc interference causes current and voltage to vary non-uniformly, leading to difficulties in seam tracking. Therefore, in this study, an optimal signal was identified for seam tracking in tandem welding and evaluated. To select the seam-tracking signal, an algorithm was developed that separates the welding signal into peak, average, and base. Based on the collected data, regression and signal-to-noise ratio analyses were performed to identify a suitable seam-tracking signal. To trace the welding line based on the selected signal, the welding signal was checked by weaving on the V-groove specimen. As a result, the current area difference of the welding signal generated between the left and right parts of the center of the V-groove could be calculated. An algorithm and equipment for seam tracking were constructed using the area difference of the welding current. Finally, the seam tracking system was verified by conducting an actual test using the equipment to which the algorithm was applied.

## Introduction

The tandem welding method utilizes two wires simultaneously and improves productivity when welding thick plates; it is extensively used in various industrial fields^1–4^. However, in this process, the arc heat source is generated from both wires; thus, the amount of heat generated is higher than that of the general gas metal arc welding (GMAW) process, leading to increased thermal deformation^5^. Therefore, to cope with the welding heat deformation, it is essential to trace the welding location. The Optical methods and methods based on arc sensing are commonly used for seam tracking^6–10^.

The optical method identifies the welding position through image processing by acquiring an image of the welding position using a vision camera after it has been irradiated with a line laser. Instead of a laser, Xu et al. traced the weld line by analyzing the gradient of the image caused by the arc light^7^. However, a disadvantage of this method is that the signal can be distorted by arc light, and additional equipment is required. In contrast, the arc sensing method facilitates more effective tracking of the welding line during the welding process regardless of the oscillation of the arc light^11^.

The welding current and voltage vary according to the contact-tip-to-work distance (CTWD), which is the distance between the base material and the contact tip located at the end of the torch; the arc sensing method uses these variations to track the welding line. In recent years, arc characteristics have been extensively investigated to identify those applicable in improving arc sensing technology in new welding processes such as cold metal transfer and keyhole-tungsten inert gas welding^12–14^. Furthermore, an algorithm that can automatically recognize the gap width during narrow gap welding was developed by applying a neural network technique^15^. Thus, the applicability of artificial intelligence in arc sensing is also being investigated.

An arc sensing technology that can analyze welding signals, such as current and voltage, is essential. In this regard, Shi et al. used a rotational arc sensor in GMAW to find the offset distance as the center point changed and conducted a study on seam tracking based on this^10^. Similarly, Shi et al. used a high-speed rotational arc sensor to analyze the signal patterns that vary according to the changes in rotation frequency, V-groove angle, and rotation diameter^16^. Le et al. performed right-angle fillet weld tracking using a robot^17^. Gao et al. performed seam tracking in curved fillet welds by detecting the welding gun angle based on the welding current^18^. Bingul et al. conducted a study to predict electrode extension in real time by detecting the change in the voltage signal in GMAW^19^. Liu et al. studied signal selection for seam tracking using arc sensing in the pulsed GMAW process; they analyzed the peak, average, base, and pulse frequency components^20^. In addition, Liu et al. studied seam tracking using arc sound^21^. Thus, several studies have investigated arc sensing.

However, the present study focuses on tandem-type welding, which uses two arc plasmas simultaneously; this method requires a different approach for arc sensing as arc interference occasionally occurs between the two wires^22^. The signal for seam tracking is affected by arc interference, making it difficult to track the welding line using the existing method. However, despite challenges, the application of arc sensors in tandem welding has not yet been thoroughly investigated.

In this study, we selected an appropriate seam-tracking signal, developed an algorithm to track the welding location based on the selected signal, and evaluated the seam-tracking performance while conducting actual tandem welding. Among the various tandem welding methods, asynchronous tandem welding, wherein the two power sources were not synchronized, was selected and applied. Because the selection of signals is limited to welding current and arc voltage, we designed and demonstrated a device that acquires current and voltage signals from each torch and analyzed the measured current and voltage signals to identify an optimal signal with high sensitivity. We performed a seam tracking test by analyzing the selected signal in real time.

## Experiment equipment configuration

### Tandem welding waveform measurement

**Figure 1:**
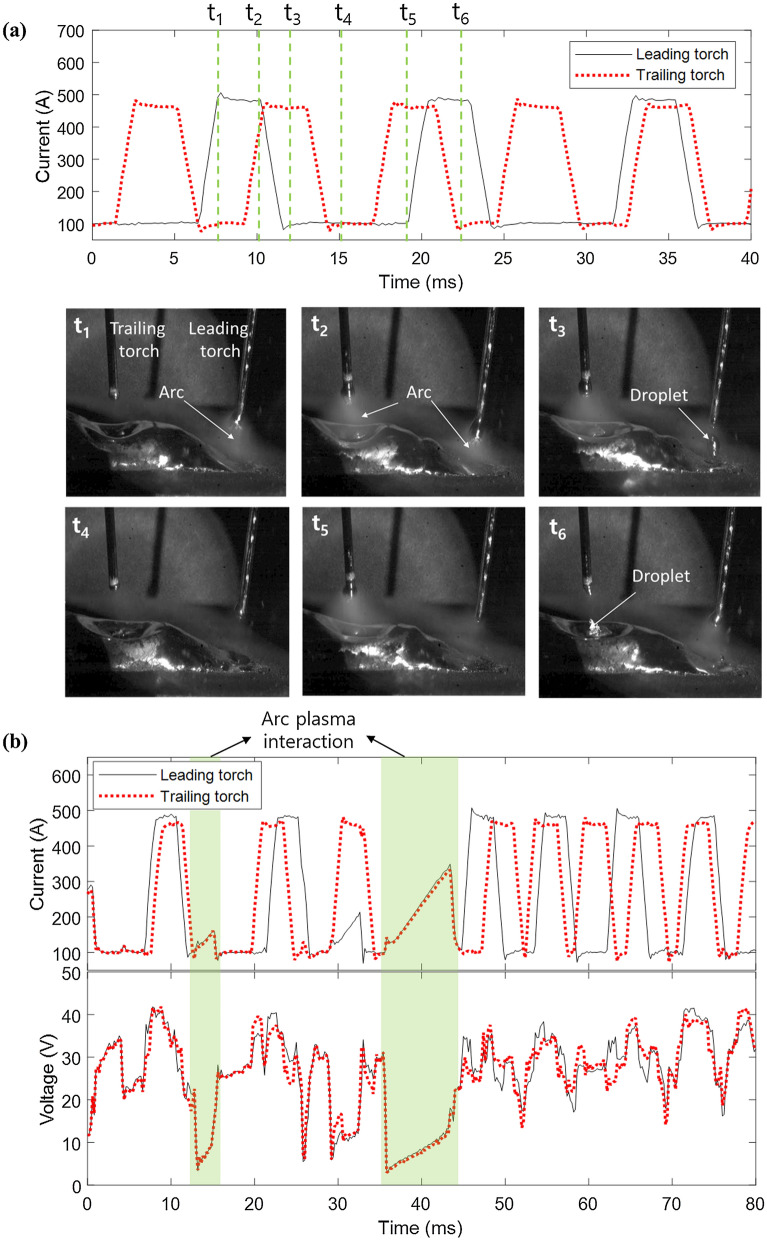
Asynchronous tandem welding mechanism and problem. (**a**) Tandem welding pulse current and molten metal transfer and (**b**) destroyed pulse current and voltage by arc interruption in tandem welding (color zone).

The welding current waveform is illustrated in Fig. 1. It is a combination of two DC pulses of the welding machine, and the phase difference between the two pulses is not synchronized. Both pulses were welded under the same conditions. Therefore, it can be confirmed that the current frequency in the trailing torch increases, as shown in the waveform in Fig. 1a. Because the CTWD in the trailing torch is shortened owing to the beads generated from the leading torch, the current frequency of the trailing torch increases, and the average current increases (Fig. 1a). At time t_1_, shown in Fig. 1a, when the leading torch rises, an arc occurs, and the metal wire melts. Subsequently, arcs are generated in both wires, at time t_2_ in Fig. 1a, and the amount of molten metal at the tip of the leading torch wire increases. As indicated in Fig. 1a, at time t_3_, the molten metal of the leading torch was applied, and that of the trailing torch was made larger and subsequently transferred. At time t_4_, both wires maintained the base current state and a minimal arc that did not melt the metal. Moreover, at time t_5_ (Fig. 1a), the current of the trailing torch increases, melting the metal. At time t_6_, the molten metal is transferred from the trailing torch to the molten pool, and the current of the leading torch is increased to melt the metal wire of the leading torch.

In this case, when the arcs are turned on simultaneously, arc interference may occur, as shown in Fig. 1b. The phase difference between the two is the same, and the waveform of the pulse is deformed. When arc interference occurs, the voltage decreases to approximately 3 V and then returns to the normal waveform. Owing to the change in the current and voltage waveforms due to such arc interference, it becomes difficult to trace the welding line.

### Welding signal measurement equipment configuration

**Figure 2:**
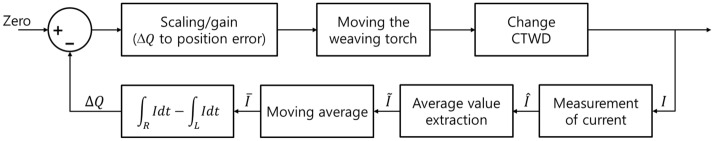
Data processing for seam tracking algorithm.

The algorithm developed and processed in real time for seam tracking is shown in Fig. 2. By measuring the current and voltage, each data point is separated into peak, average, and base, and the deviation of the data is reduced by using a moving average. The signal that responds most sensitively to the arc length was selected to select the most appropriate signal for welding line tracking. In the V-groove specimen, the area difference ($\Delta Q$) between the left and right signals at the center of the specimen was calculated. Finally, the welding line was traced after the motor feed value was determined based on the calculated value.

**Figure 3:**
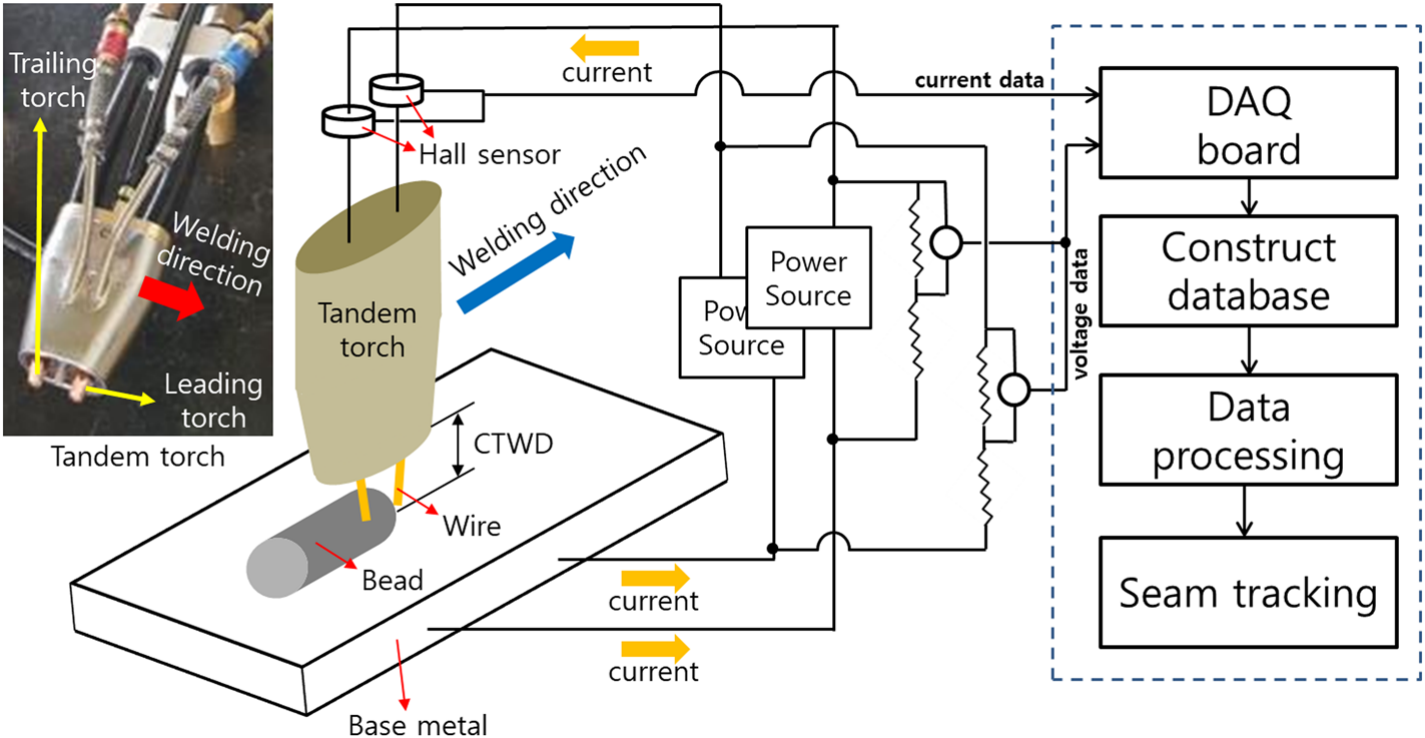
Measuring welding signal and seam tracking system schematic. Components: welding power source, hall sensor (PAC-22), voltage divider, and data acquisition (DAQ) board (NI-9221). *CTWD* contact-to-work distance.

The equipment for tracking the welding line by measuring the pulse welding signal and analyzing the data was configured as shown in Fig. 3. The device comprises a tandem torch, Hall sensor, voltage divider, data acquisition (DAQ) board for data collection, and two welding power supplies. A Hall sensor and voltage divider were configured for each welding power source. To reduce the voltage according to the specifications of the DAQ board, a voltage divider that reduces the voltage by 16 times was used. The sampling frequency of the DAQ board was 50 kHz. An algorithm was constructed to correct the collected data and pre-process the data, and the welding line was traced through feedback.

**Figure 4:**
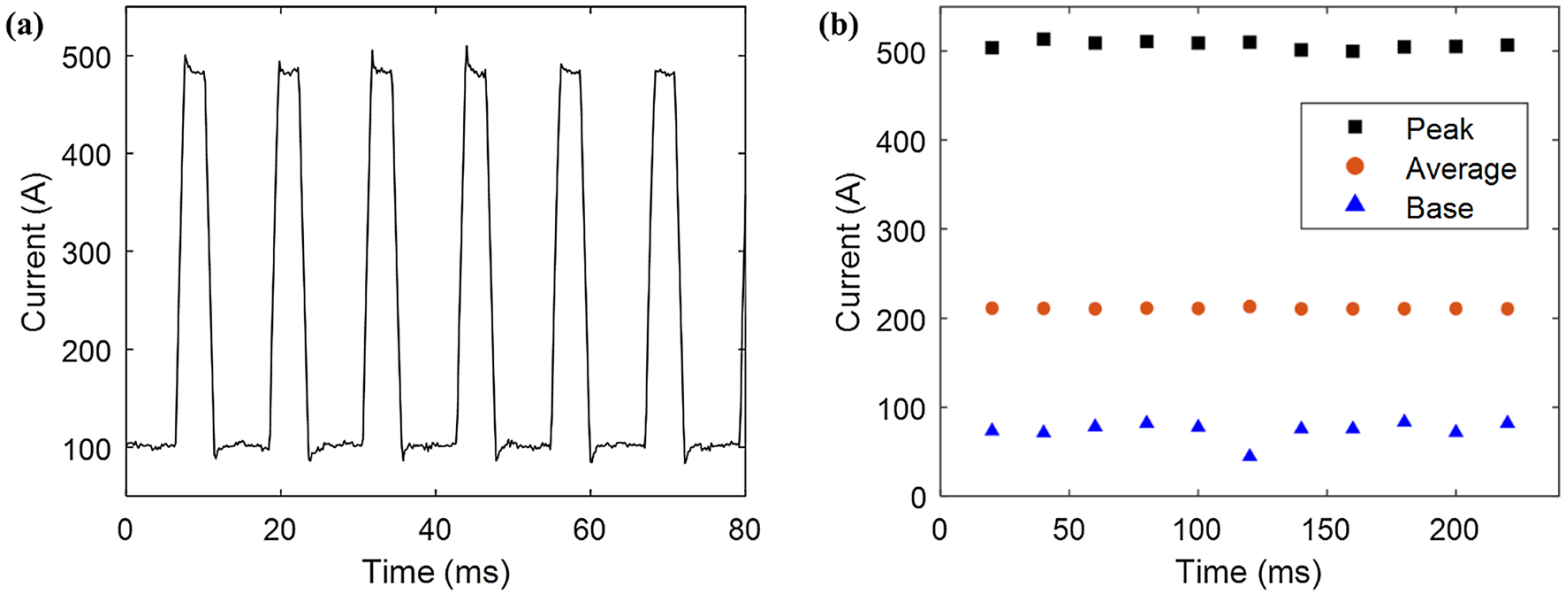
Data separation process. (**a**) Raw data of welding current and (**b**) current data with moving average applied after separating into peak, base, and average (wire feeding speed: 6 m/min, contact-to-work distance: 16 mm).

The data separation process is shown in Fig. 4. Figure 4a shows the raw data of the welding pulse current. The collected data are divided into peak, average, and base currents every 20 ms. Current/voltage signals were measured for 20 s, and a total of 3000 data points (peak, average, and base) were collected per welding condition. For the measured data, the deviation was minimized using the moving average method, reducing the non-uniformity of the current and voltage signals due to arc interference. This is illustrated in Fig. 4b. Both the leading and trailing torches proceeded in the same way; the voltage was separated, and data were collected.

## Seam tracking signal analysis

### Signal selection for seam tracking in tandem welding

**Figure 5:**
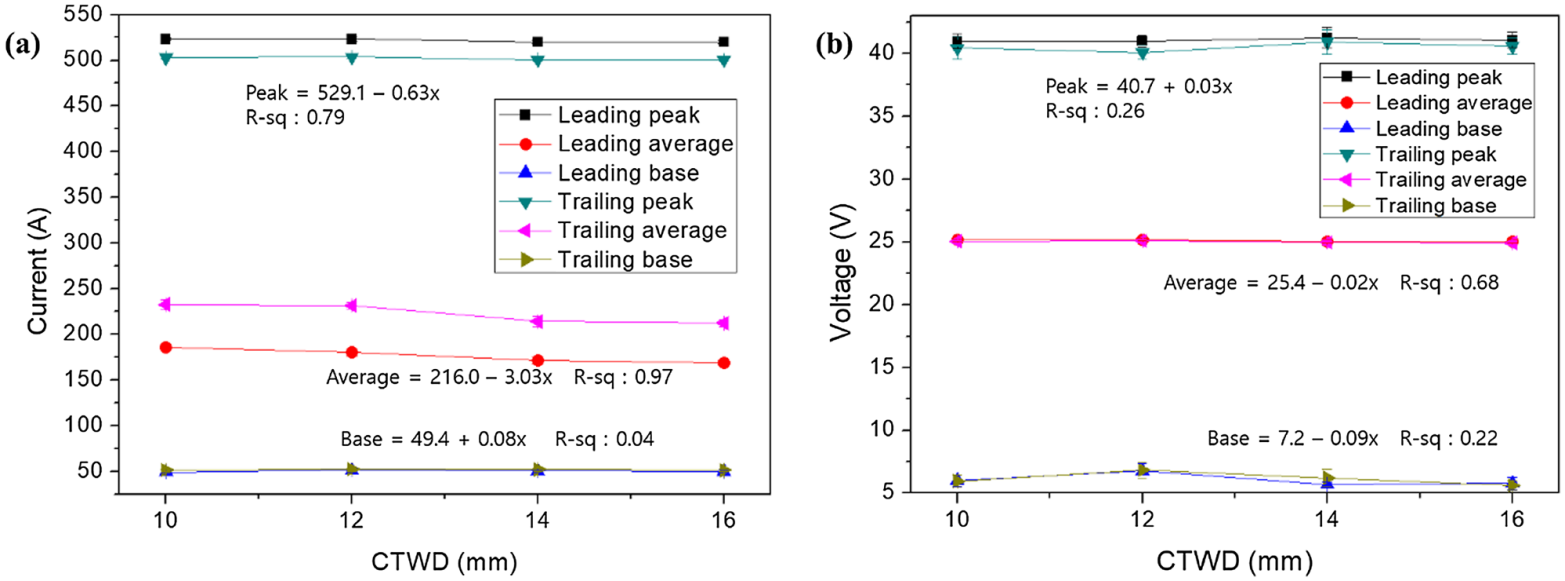
Regression analysis for seam tracking. (**a**) Current and (**b**) voltage variation with contact-to-work distance (CTWD) for wire feeding speed of 6 m/min.

The experiment was conducted under the following conditions: Wire feed speed (WFS) was tested at 6 and 8 m/min under the same conditions for both leading and trailing torches. The welding current depends on the WFS. For WFS of 6 and 8 m/min, the welding current was 180 and 230 A, respectively. The experiment was conducted using the bead-on-plate method. The welding speed was fixed at 60 cm/min, and Ar 80% + CO_2_ 20% was used as the shielding gas. The CTWD was tested by increasing it from 10 to 18 mm in 2 mm increments. Figure 5 shows the current and voltage signals of the leading and trailing torch when the WFS is 6 m/min (see Supplementary data 1, 2). Through regression analysis, the current and voltage signals that were most sensitive to CTWD changes were identified. As shown in Fig. 5a, the average current of the trailing torch is higher than that of the leading torch; this phenomenon can be attributed to the short pulse period of the trailing torch. The slope of the average current in Fig. 5a is approximately 3, and the slope of the average voltage in Fig. 5b is 0.02, confirming that the average current has a higher sensitivity than all other signals. In addition, the base voltage is unsuitable as a seam-tracking signal because a significant deviation is induced due to arc interference and short circuit. To check the linearity of the signal change, *R*^2^ was compared, and the *R*^2^ of the average current was the highest at 97%. Therefore, the average-current signal is the most appropriate for tracing the welding line.

**Figure 6:**
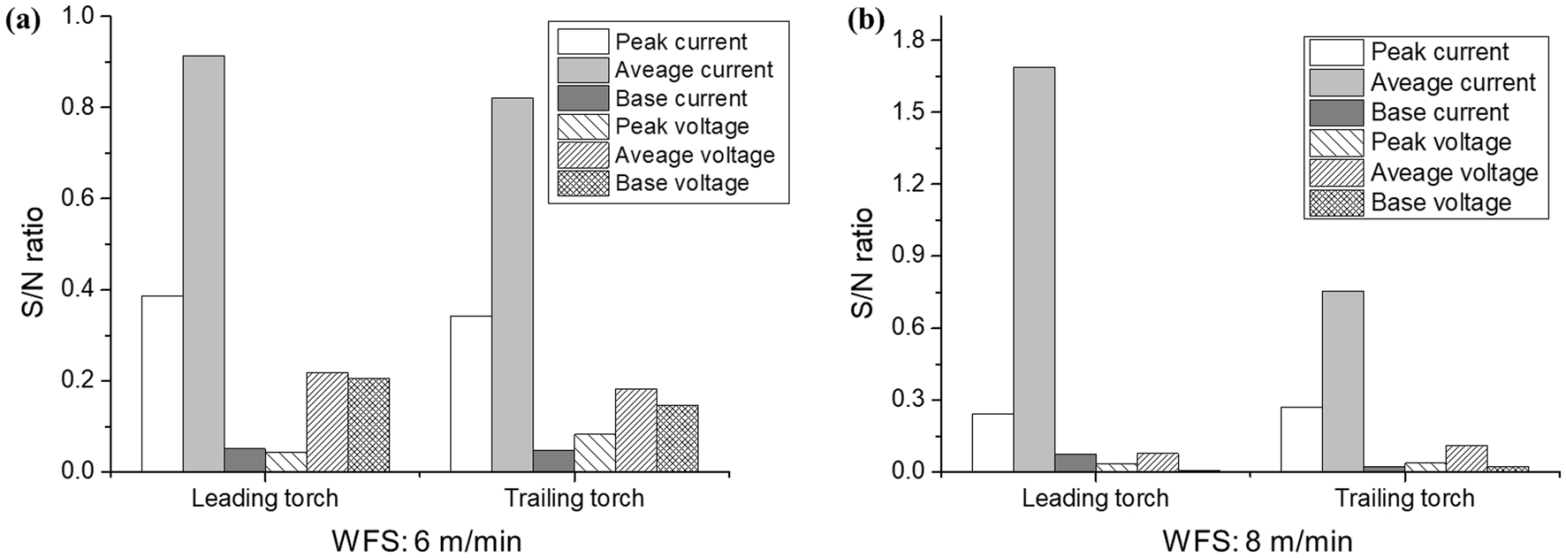
S/N ratio analysis for seam tracking signal selection for wire feeding speed (WFS) of (**a**) 6 m/min and (**b**) 8 m/min.

To analyze the sensitivity of the signal more accurately, the S/N ratio was calculated, as shown in Fig. 6 (see Supplemetary data 3). The S/N ratio was calculated as *G*/*σ*, where *G* is the slope of the signal, and *σ* is its standard deviation^23^. When comparing the peak, base, and average signals, the average signal exhibited the best S/N ratio among all. The S/N ratio for the voltage signal was mostly low, and the value with the highest S/N ratio was 0.22. Among the current signals, the base and average currents had the lowest and highest S/N ratio, respectively. In addition, it can be observed that most of the signals from the leading torch have a higher S/N ratio than those from the trailing torch. This is because the CTWD and arc length of the trailing torch become unstable owing to the behavior of the molten pool generated by the leading torch, increasing the resulting deviation. As the deviation increased, the signal-to-noise (S/N) ratio of the trailing torch decreased as a whole. Therefore, the most appropriate signal for the welding line tracking signal is the average current signal of the leading torch with the largest S/N ratio.

### Seam tracking algorithm creation

**Figure 7:**
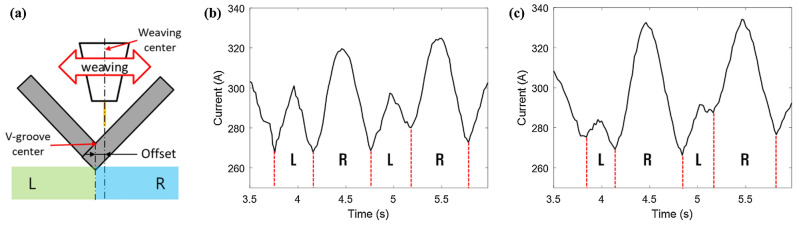
Area difference between the L area and R area during weaving. (**a**) Experiment schematic; (**b**) offset = 1 mm (area difference = 3000); (**c**) offset = 2 mm (area difference = 6000).

To secure the data for seam tracking, the experiment was conducted by weaving the V-shaped specimen, as shown in Fig. 7a. The amplitude of weaving was fixed as 6 mm, and the distances between the center of the weave and that of the V-shaped specimen were fixed as 1 and 2 mm, respectively. The experimental results are presented in Fig. 7b,c (see Supplementary data 4). The minimum values shown in Fig. 7b,c are formed at the center of the V-groove (Fig. 7a). Therefore, the area difference (charge difference) between the left and right welding currents can be calculated as follows:

1 $$\text{Area difference}(\text{charge difference})={\int }_{R}I dt-{\int }_{L}I dt.$$

Based on the calculated area difference of the welding current, if the area difference of the welding current is negative, the center of weaving is to the left of the center of the V-groove; if the area difference is positive, the center of the weaving is to the right. Subsequently, the position correction value of the motor can be extracted by calculating the linear relationship between the area difference of the welding current and the offset. As shown in Fig. 7b, when the offset is 1, the area difference of current is approximately 3000, and when the offset is 2, as shown in Fig. 7c, the area difference of current is approximately 6000. Based on this, the area difference is used to calculate the offset.

## Seam tracking validation

**Figure 8:**
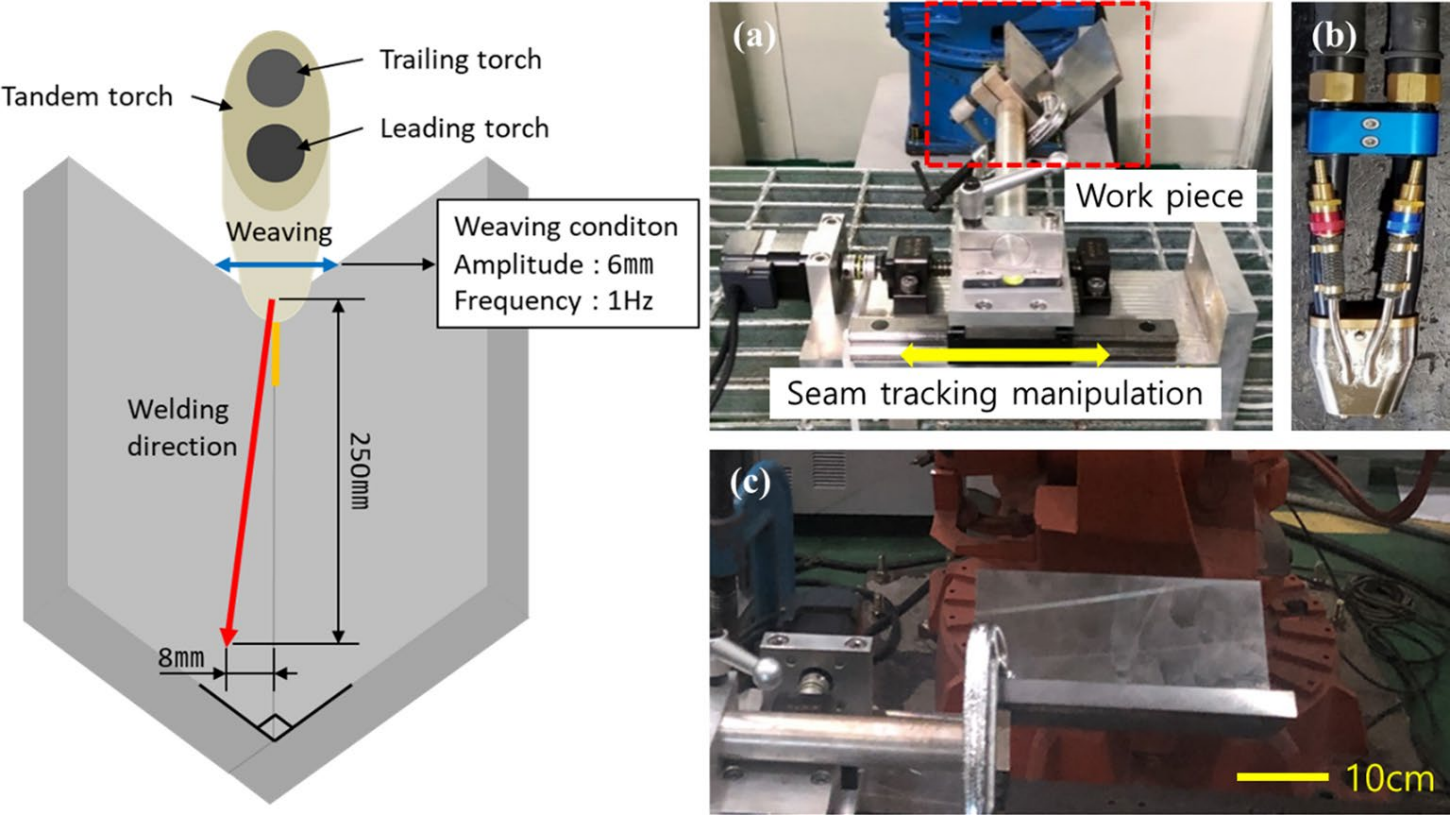
Welding method and device configuration for seam tracking. (**a**) Experimental schematic for welding line trace test, (**b**) seam tracking device configuration, (**c**) tandem torch, and (**d**) base material.

The seam-tracking verification experiment was conducted based on previously selected data and the motor feed value for tracking the welding line. The experimental method and equipment were configured as shown in Fig. 8. Welding was started by setting the distance between the center of the weaving and the center of the V-groove to match. The test was carried out at a distance of 8 mm from the welding endpoint. The weaving frequency was set to 1 Hz, and the amplitude of the weaving was set to 6 mm. A single-axis servo motor was used for seam tracking, and a motor control algorithm for seam tracking was produced using LabVIEW 2017. The measurement system (Fig. 3) and the algorithm for calculating the current area difference (Fig. 7) and motor position correction value were configured in LabVIEW. In the weaving cycle, the motor position correction value was calculated once per second, and the motor was controlled based on the calculated value. A seam tracking test was conducted using the built-in equipment and algorithm.

**Figure 9:**
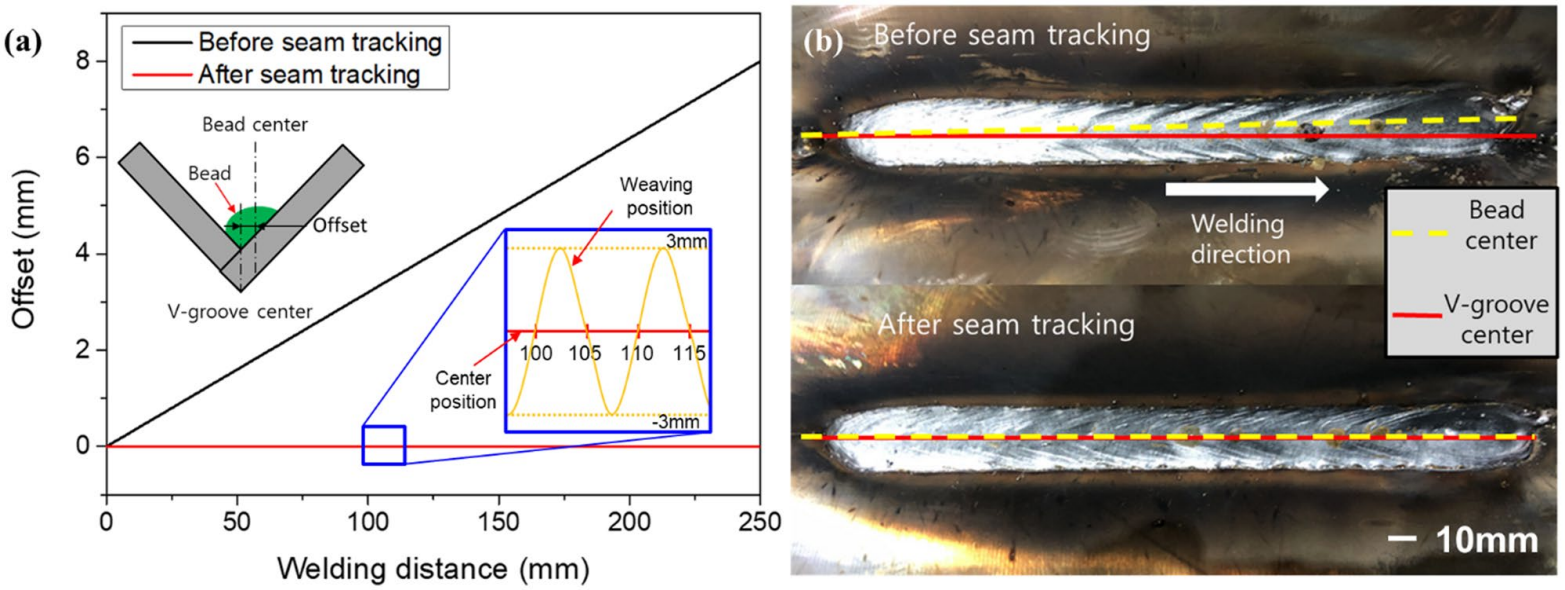
Comparison of results before and after seam tracking. (**a**) Offset between the center of the bead and the center of the V-groove appearing before and after applying the seam tracking. (**b**) Seam tracking results.

The seam-tracking results are presented in Fig. 9. Before applying the weld line tracking, the bead exhibited a tendency to move away from the center of the V-groove material. As shown in Fig. 9a, an error of approximately 8 mm occurred at the point where the welding ended. By applying the welding line tracking algorithm, it can be confirmed that the center of the bead and the center of the V-groove coincide.

## Conclusion

A seam tracking system was established to prevent quality deterioration due to thermal deformation during tandem welding. Arc sensing is difficult due to arc interference in the existing seam tracking method. The present study proposed a new algorithm and seam tracking system to minimize arc interference. To build the seam tracking system, the most sensitive signal was selected according to CTWD, and based on the selected signal, a welding line tracking system was developed and tested.

The current and voltage signals vary irregularly due to arc interference. The current/voltage of the leading and trailing torches were measured by establishing a monitoring system and separated into peak, base, and average. To select the most sensitive signal among these, the variations in current and voltage according to changes in CTWD were compared, and the slope of the average current was the highest at approximately 3. Also, the *R*^2^ of the average current was the highest at 97%, and the linearity was the highest. The S/N ratio was calculated based on the slope and standard deviation, and the average current, with the highest S/N ratio, was selected as the most appropriate signal for seam tracking.

To apply the selected signal to seam tracking, the signal was measured by weaving the V-groove specimen. The test was conducted while offsetting the center of the V-groove specimen and the center of weaving. The change in average current was different when the offset was 1 mm and 2 mm, and the offset was calculated based on the area difference of welding current. A linear relationship between the area difference of welding current and offset was established and applied to the seam tracking system. The weld bead coincided with the center of the V-groove specimen in the case of welding with the application of seam tracking. Therefore, seam tracking can be performed using the area difference of welding current, and the method has the potential for application in the welding of large structures.

## Supplementary Information

Supplementary Information.

## Data Availability

The datasets used and/or analyzed during the current study are available from the corresponding author upon reasonable request.
